# Global clues to the nature of genomic mutations in humans

**DOI:** 10.7554/eLife.27605

**Published:** 2017-05-17

**Authors:** Aylwyn Scally

**Affiliations:** Department of Genetics, University of Cambridge, Cambridge, United Kingdoma.scally@gen.cam.ac.uk

**Keywords:** population genetics, mutagenesis, great ape evolution, DNA replication and repair, mutational signatures, human population structure, Human

## Abstract

An analysis of worldwide human genetic variation reveals the footprints of ancient changes in genomic mutation processes.

**Related research article** Harris K, Pritchard JK. 2017. Rapid evolution of the human mutation spectrum. *eLife* **6**:e24284. doi: 10.7554/eLife.24284

All children inherit a mixture of chromosomal sequences from their parents, and although the copying process involved is extremely accurate, some errors occur. We refer to such errors as de novo (new) germline mutations, and when they are passed on to subsequent generations, these mutations are the raw material on which natural selection works and the source of all genetic differences between populations and species. Most have negligible or minor effects, but some on very rare occasions are responsible for serious genetic disease (Veltman and Brunner, 2012). The accumulation of genetic differences through mutation is also a primary source of information about human evolution. Thus it is important to understand the nature of genomic mutation, the rate at which it occurs and the factors causing it.

A key question is to what extent mutation processes differ between individuals, either in the total number of de novo mutations bequeathed to offspring or the places in the genome where they occur. Such differences could be genetic in origin, given that the proteins involved in DNA replication are themselves encoded in the genome, and there could also be environmental effects associated with where and how an individual lives. In both cases these factors may reflect recent evolutionary events, particularly the divergence of human populations and their global dispersal within the last 100,000 years.

One way to address this question would be to collect genomic and other data for thousands of families, identify de novo mutations in each of the offspring, and then analyse the factors contributing to them. However, this approach is extremely demanding in terms of the resources needed. Now, in eLife, Kelley Harris and Jonathan Pritchard of Stanford University report how they have taken an alternative approach (Harris and Pritchard, 2017) that involved using a dataset of whole-genome sequences for 2504 individuals from 26 different populations around the world (Auton et al., 2015). Consider that any genetic variant in this dataset, even if it is found today in many individuals, was once a de novo mutation in a single ancestor. Thus if different genetic or environmental factors have affected mutation processes in different human populations, we might expect to find evidence in the distribution of variants in these populations today.

Harris and Pritchard categorised single-nucleotide variants in this dataset by their ancestral and derived alleles (i.e. the version before and after mutation) and their sequence context as represented by the two flanking nucleotides. For example, the category AGC → ATC represents a mutation from G to T with flanking nucleotides A and C. After counting the number of variants in each category within every individual, the researchers found that the distribution of counts, termed the mutation spectrum, differs between populations to the extent that it is possible to identify an individual’s continent of origin based solely on the spectrum of mutations they carry. In general the differences between spectra comprise a multitude of small discrepancies, rather than large discrepancies in a few categories. However some categories do stand out, most notably an increased abundance of TCC → TTC mutations in European and South Asian populations – a signal also seen in other recent studies based on similar data (Mathieson and Reich, 2017; Narasimhan et al., 2016).

Harris and Pritchard then looked at how this signal changes with time. Mutations themselves have no timestamp, but on average a mutation that is rare in the population is likely to have arisen more recently than a mutation that is common. Using frequency as a proxy for age, Harris and Pritchard found that the enrichment for TCC → TTC was evident mainly in variants of intermediate age, and not in recent or very old variants. The data fit a model in which there was a pulse of mutation between about 2,000 and 15,000 years ago in the ancestors of present-day Europeans and South Asians, during which these mutations were 30–40% more likely.

One possible explanation is that this is the legacy of a mutator allele (that is, a mutation that increases the rate of de novo mutation in some or all categories) that appeared and survived in the population for several thousand years before going extinct (Figure 1). It is not known how frequently such alleles arise, or to what extent they drive differences in the mutation spectrum: however it does seem that under certain conditions they can survive for a long time and have lasting effects (Seoighe and Scally, 2017). In addition to a mutator allele that would have increased the relative rate of the TCC → TTC mutation, it is possible that other mutator alleles that had smaller effects and/or survived for shorter times in different populations may be responsible for less prominent differences in the mutation spectrum.

**Figure 1.**
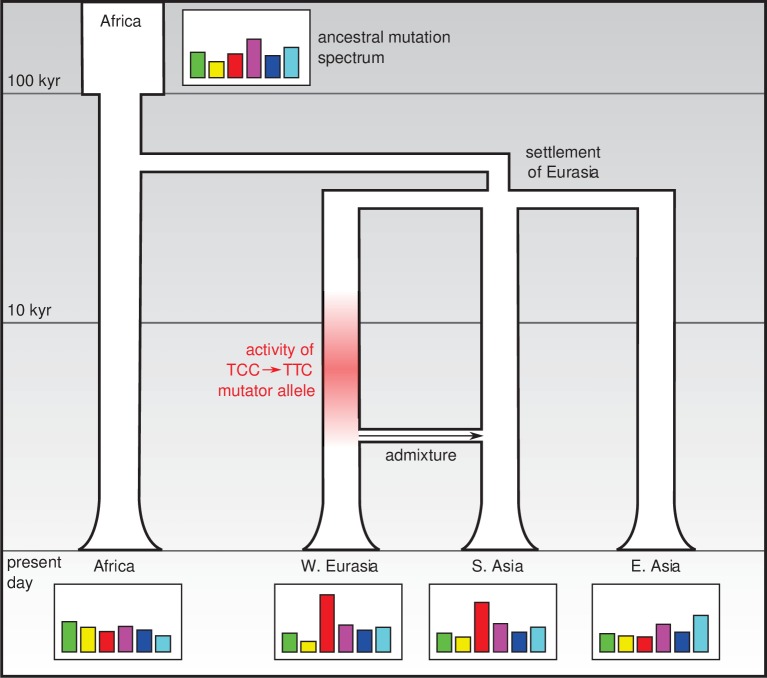
Changes in the genetic mutation spectrum in humans. Modern humans originated in Africa (top left) and spread into Eurasia in a series of migrations 50,000–80,000 years ago. Subsequent migrations within Eurasia (such as the migration between West Eurasia and South Asia around 3000 years ago that is shown here; Reich et al., 2009) led to secondary genetic contact and admixture. The mutation spectrum (represented abstractly here) evolved separately in each population after divergence, perhaps due to the effect of mutator alleles: Harris and Pritchard propose that such a mutator allele could have been responsible for an increase in TCC → TTC mutations between 2,000 and 15,000 years ago (red shading) which influenced the mutation spectra of present-day Europeans and South Asians.

These findings are also relevant to the question of whether or not the overall rate of genomic mutation varies between populations and over time. There is evidence that the mean rate has probably not changed in at least the last 50,000 years (Fu et al., 2014), but we also know that the genome-wide mutation rate has slowed down when measured over timescales of millions of years (Moorjani et al., 2016). The results of Harris and Pritchard involve the relative rates of mutation in different sequence contexts, and so strictly speaking they do not tell us about variation in the overall rate, but they are suggestive of differences perhaps on the order of a few percent.

The next few years will see a substantial increase in the amount of available de novo mutation data, particularly from large-scale sequencing projects aimed at understanding the causes of rare genetic disease. These data will enable direct exploration of the factors determining mutation and its evolution within human populations, and shed further light on the questions addressed and raised by Harris and Pritchard.

## References

[bib1] Auton A, Brooks LD, Durbin RM, Garrison EP, Kang HM, Korbel JO, Marchini JL, McCarthy S, McVean GA, Abecasis GR, The 1000 Genomes Project Consortium (2015). A global reference for human genetic variation. Nature.

[bib2] Fu Q, Li H, Moorjani P, Jay F, Slepchenko SM, Bondarev AA, Johnson PLF, Aximu-Petri A, Prüfer K, de Filippo C, Meyer M, Zwyns N, Salazar-García DC, Kuzmin YV, Keates SG, Kosintsev PA, Razhev DI, Richards MP, Peristov NV, Lachmann M, Douka K, Higham TF, Slatkin M, Hublin JJ, Reich D, Kelso J, Viola TB, Pääbo S (2014). Genome sequence of a 45,000-year-old modern human from western Siberia. Nature.

[bib3] Harris K, Pritchard JK (2017). Rapid evolution of the human mutation spectrum. eLife.

[bib4] Mathieson I, Reich D (2017). Differences in the rare variant spectrum among human populations. PLoS Genetics.

[bib5] Moorjani P, Amorim CEG, Arndt PF, Przeworski M (2016). Variation in the molecular clock of primates. PNAS.

[bib6] Narasimhan VM, Rahbari R, Scally A, Wuster A, Mason D, Xue Y, Wright J, Trembath RC, Maher ER, van Heel DA, Auton A, Hurles ME, Tyler-Smith C, Durbin R (2016). A direct multi-generational estimate of the human mutation rate from autozygous segments seen in thousands of parentally related individuals. bioRxiv.

[bib7] Reich D, Thangaraj K, Patterson N, Price AL, Singh L (2009). Reconstructing Indian population history. Nature.

[bib8] Seoighe C, Scally A (2017). Inference of candidate germline mutator loci in humans from genome-wide haplotype data. PLoS Genetics.

[bib9] Veltman JA, Brunner HG (2012). *De novo* mutations in human genetic disease. Nature Reviews Genetics.

